# Three-Dimensional Volumetric Changes and Clinical Outcomes after Decompression with DIAM™ Implantation in Patients with Degenerative Lumbar Spine Diseases

**DOI:** 10.3390/medicina56120723

**Published:** 2020-12-21

**Authors:** Cheng-Yu Li, Mao-Yu Chen, Chen-Nen Chang, Jiun-Lin Yan

**Affiliations:** 1 Department of Neurosurgery, Chang Gung Memorial Hospital, Linkou Medical Center, Chang Gung University, Taoyuan 33302, Taiwan; mick791212@hotmail.com (C.-Y.L.); justin@mail.cgu.edu.tw (C.-N.C.); 2 Department of Neurosurgery, Keelung Chang Gung Memorial Hospital, Keelung 20401, Taiwan; mailtomaxi@gmail.com; 3 College of Medicine, Chang Gung University, Taoyuan 33302, Taiwan

**Keywords:** DIAM, interspinous process device, decompression, degenerative lumbar spine diseases, magnetic resonance imaging, three-dimensional

## Abstract

*Background and objectives*: The prevalence of degenerative lumbar spine diseases has increased. In addition to standard lumbar decompression and/or fusion techniques, implantation of interspinous process devices (IPDs) can provide clinical benefits in highly selected patients. However, changes in spinal structures after IPD implantation using magnetic resonance imaging (MRI) have rarely been discussed. This volumetric study aimed to evaluate the effect of IPD implantation on the intervertebral disc and foramen using three-dimensional assessment. *Materials and Methods*: We retrospectively reviewed patients with lumbar degenerative disc diseases treated with IPD implantation and foraminotomy and/or discectomy between January 2016 and December 2019. The mean follow-up period was 13.6 months. The perioperative lumbar MRI data were processed for 3D-volumetric analysis. Clinical outcomes, including the Prolo scale and visual analog scale (VAS) scores, and radiographic outcomes, such as the disc height, foraminal area, and translation, were analyzed. *Results*: Fifty patients were included in our study. At the one-year follow-up, the VAS and Prolo scale scores significantly improved (both *p* < 0.001). The disc height and foraminal area on radiographs also increased significantly, but with limited effects up to three months postoperatively. MRI revealed an increased postoperative disc height with a mean difference of 0.5 ± 0.1 mm (*p* < 0.001). Although the mean disc volume difference did not significantly increase, the mean foraminal volume difference was 0.4 ± 0.16 mm^3^ (*p* < 0.05). *Conclusions*: In select patients with degenerative disc diseases or lumbar spinal stenosis, the intervertebral foramen was enlarged, and disc loading was reduced after IPD implantation with decompression surgery. The 3D findings were compatible with the clinical benefits.

## 1. Introduction

The prevalence of degenerative lumbar spine diseases, including disc degeneration, spinal stenosis, and dynamic instability, has significantly increased over the past few years. Ravindra et al. reported that 266 million individuals (3.63%) worldwide have degenerative lumbar spine diseases, while 403 million (5.5%) individuals worldwide were found with symptomatic disc degeneration [1]. Most of these diseases can be treated conservatively; however, when nonsurgical treatment fails, operations are then required for symptom relief. Decompression surgery with or without standard lumbar fusion techniques has been the most common surgical treatment for these disease entities. In recent years, lumbar interspinous process device (IPD) implantation has gradually drawn attention as a nonfusion technique after decompression surgery [2,3]. The goal of IPD implantation includes restoring the disc height, decreasing posterior element destruction, and reserving micromotion after adequate decompression under minimally invasive techniques [4,5]. Current studies have suggested that these instruments can provide good clinical outcomes and reduce adjacent segment degeneration when combined with fusion operations, except in the case of degenerative spondylolisthesis [6,7]. In addition, two recent meta-analyses also reported that IPD implanted showed similar postoperative outcomes to traditional fusion techniques in highly selected patients [6,8].

On the other hand, some controversies of IPDs have been mentioned in other systemic reviews and meta-analyses [9,10,11]. These studies have shown high reoperation rate and unsustainable clinical improvements after IPDs implantation. However, there are several common limitations in these studies. First, surgical techniques were mostly percutaneous IPD implantation, which indicated that they did not provide adequate decompression. In addition, these studies used relative fixed device, rather than elastic materials. Our study, consequently, emphasizes on the combination of IPD implantation after adequate decompression (foraminotomy/laminotomy).

Among various types of IPDs, implantation of the device for intervertebral assisted motion (DIAM, Medtronic, Inc., Memphis, TN, USA) appears to create similar intradiscal pressure to that of the intact model because of its lower rigidity and softer texture [8]. Its effects in increasing disc height and decompression are also similar to those of other IPDs. Previous studies have demonstrated its clinical benefits, with multiple mechanisms being discussed [4,12,13]. However, most of these studies were performed in vitro. Due to limitations of the health care system, insurance, and costs, volumetric changes after DIAM implantation with decompression surgery in MRI have not been established. Whether the three-dimensional effects created by DIAM correlate with its clinical symptoms also remain unclear. Therefore, the aim of our study was to analyze the effects of DIAM implantation on volume through three-dimensional MRI measurements and to evaluate whether these effects are correlated with radiographic or clinical outcomes.

## 2. Materials and Methods

We retrospectively reviewed the medical records of patients who had received lumbar decompression with DIAM^TM^ implantation from January 2016 to December 2019 in a single medical center. The study was approved by our institutional review board (IRB: 202001153B0). Written informed consent was obtained from patients who participated in the study. The inclusion criteria were persistent axial low back pain, leg pain, or intermittent claudication caused by degenerative disc diseases (DDDs) and/or lumbar canal stenosis, foraminal stenosis, grade I spondylolisthesis, and herniated discs after 12 weeks of conservative treatment, which included lifestyle adjustments, pain relief medications, and physical therapy. We excluded patients with pars fractures, segmental instability (grade II spondylolisthesis and above), sequestered foraminal discs, disc space collapse with advanced disc degeneration (Pfirrmann grades IV and V), and combined hip joint degeneration. The operation was performed by two neurosurgeons who both had five years of experience with the procedure.

### 2.1. Patient Characteristics

The patient demographics and preoperative conditions are shown in Table 1. A total of 50 patients, 13 males and 37 females, were included in our study, with a mean age of 62.1 years old. Most of our patients had multiple complaints, with 44 patients complaining of low back pain, 43 with leg pain with or without intermittent claudication, and 13 with soreness. Moreover, multiple diseases on multiple levels were also seen on preoperative imaging, with 38 cases of lumbar stenosis, 29 of grade I spondylolisthesis, and 15 of herniated disc disease. DIAM was implanted after decompression at L2–3 in 4 patients, L3–4 in 2 patients, and L4–5 in 24 patients. A double-level implant procedure was performed in 18 patients: 3 at L2–3 and L3–4 and 15 at L3–4 and L4–5. A three-level procedure was performed in two patients.

### 2.2. Surgical Technique

All operations were performed under general anesthesia. Microscopic foraminotomy and/or discectomy were performed in all patients unilaterally or bilaterally according to the preoperative clinical assessment. After adequate decompression, DIAM implantation was performed using a standardized approach, with preservation of the supraspinous ligament and placement of the prosthesis as anterior as possible between the targeted spinous processes and laminae. The artificial ligaments were tightly anchored in all patients. For patients with herniated intervertebral discs, additional discectomy was performed prior to IPD implantation.

### 2.3. Clinical Outcomes and Radiographic Parameters

We evaluated our patients at a regular interval of 3 months postoperatively for 1 year. The severity of low back pain and leg pain was assessed using the visual analog scale (VAS), while simplified quality of life and functional impairment were evaluated using the Prolo scale on chart review. The Prolo scale is a widely used assessment tool for lumbar spinal surgery [14]. It acts as a secondary outcome measurement that includes both functional and economic factors to evaluate quality of life.

Lateral view and flexion-extension lumbar standing radiographs were obtained preoperatively and at each follow-up time point for bony structure assessment. Disc height (DH) was defined as the average distance between the endplates of the vertebral bodies at the DIAM-implanted level on the anterior and posterior body lines. The same method was also utilized for MRI disc height measurement. The parameters of the intervertebral foramen including the foraminal length (FL), width (FW), area and extension foraminal length (EFL), width (EFW), and area were measured at the mid-pedicular level from the superior pedicle wall to the inferior pedicle wall using the concept modified from Shin et al. [15]. We used the most commonly accepted Meyerding technique to evaluate the degree of translation. Images were analyzed by an independent neuroradiology specialist and neurosurgical resident, both blinded to the outcome.

### 2.4. 3D MRI for Volume Assessmentt

**Figure 1:**
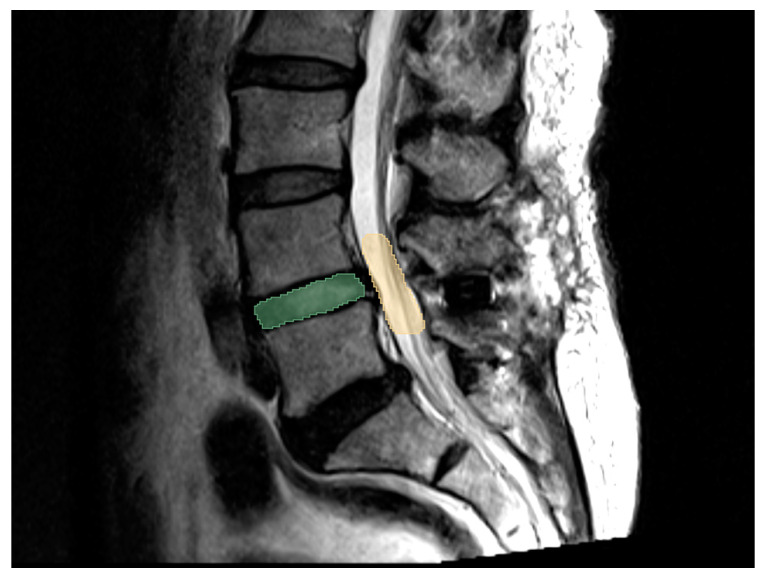
Three-dimensional volume measurement of Magnetic Resonance Imaging (MRI) via two-dimensional reconstruction sagittal image. Disc volume was measured between vertebral endplates as green area. Foraminal volume was measured between midportion of pedicles at adjacent levels as yellow area.

MRI was applied to evaluate the condition of neural structure compression: once during the preoperative period and again 1 year after the operation. Image data obtained in DICOM format were loaded onto MRIcron (Funding: NIH-NIDCD R01DC009571, McCausland Center for Brain Imaging, University of South Carolina) and transferred to NlFTI format under maximum intensity projection. These images were then aligned perpendicularly in all three dimensions (axial, coronal, and sagittal) before calculation. We chose T2-weighted images for evaluation because they provide better cerebral spinal fluid and neural structure information. Disc volume measurements, since each disc level only contained 3 axial cuts, were generally based on sagittal-viewed images of the area between the superior and inferior endplates of the vertebral bodies. This study followed the pedicle-to-pedicle technique proposed by Rao et al. [16] for foraminal volume, which was then remeasured from all three dimensions to minimize potential bias. The methods used for volume assessments are shown in Figure 1. The measured values were generated and calculated using the image software 3D slicer 4.10.2.

### 2.5. Statistical Analysis

All statistical analyses were performed using MedCalc^®^ Statistical Software version 19.6.1 (MedCalc Software Ltd., Ostend, Belgium; https://www.medcalc.org; 2020). Clinical outcomes were compared using paired *t*-tests. ANOVA or Kruskal-Wallis tests were also used to compare radiographic outcomes. A *p* value < 0.05 was regarded as significant.

## 3. Results

### 3.1. Clinical Outcomes

**Figure 2:**
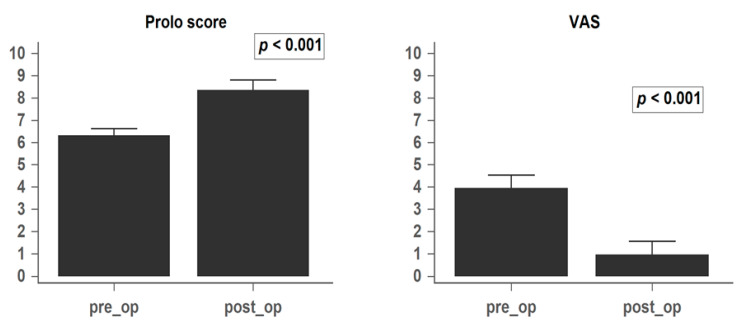
Preoperative and 1-year postoperative mean Prolo Scale and VAS (Visual Analog Scale) levels. Data are presented as mean ± SD. SD: standard deviation.

The patient clinical outcomes and recurrence rates are summarized in Table 2. DIAM implantation significantly relieved the symptoms of low back pain (improvement rate: 75%) and leg pain with or without neurogenic intermittent claudication (improvement rate: 90.7%) in our patient group from baseline to the 1-year follow-up (*p* < 0.001). The mean preoperative VAS and Prolo scale scores were 3.9 ± 1.7 and 6.3 ± 0.9, respectively, while the values at the 1-year follow-up were 0.9 ± 1.8 and 8.3 ± 1.4, respectively (*p* < 0.001; Figure 2). This indicated that the functional status and quality of life also improved. However, patients who complained of low back soreness mostly did not benefit from the surgery, with only 1 patient (7%) remaining free of symptoms.

Four patients had recurrence of low back pain/leg pain or intermittent claudication at the last follow-up despite initial improvements. The first patient had a new-onset trauma history with a pars fracture at the DIAM-implanted level, while the others showed no significant abnormalities on MRI studies. Laminectomy with fusion and fixation was performed for the first patient. The symptoms of the 3 aforementioned patients subsided after medications, physical therapy, or nerve block under arthrography.

No fracture of the spinous process or device dislocation was noted after the operation during our follow-up. No newly developed neurologic deficits were reported. The mean operative blood loss was 98 mL. The mean operative time was 176.5 min.

### 3.2. Radiographic Outcome

**Figure 3:**
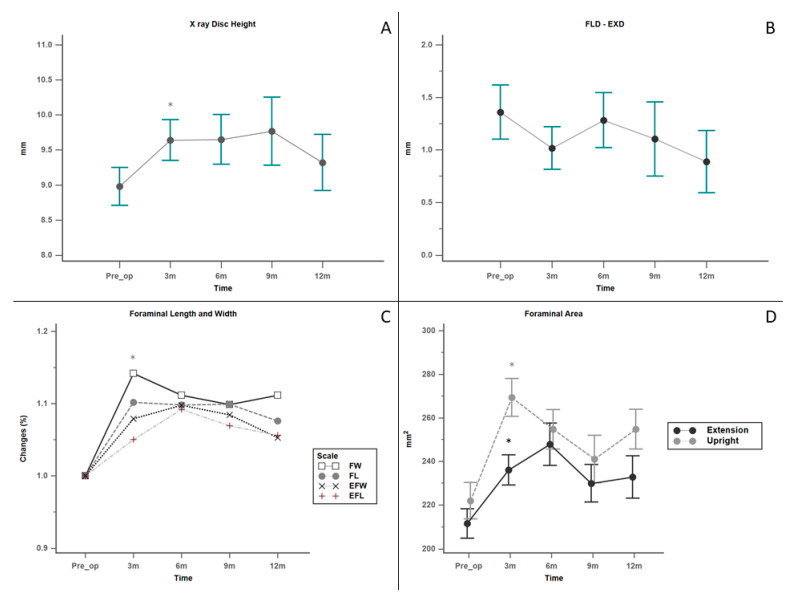
Radiographic outcomes. Values are presented as mean ± standard deviation. (**A**) Disc height (**B**) Slippage difference between flexion and extension (**C**) Foraminal Length and Width (**D**) Foraminal area * *p* < 0.05. Abbreviations: FL = Foraminal length; FW = Foraminal Width; EFL = Extension foraminal length; EFW = Extension foraminal width.

The radiographic findings are shown in Figure 3. The disc height of the DIAM-implanted segment increased significantly on follow-up radiographs (*p* < 0.05). The most prominent change was observed 3 months postoperatively (Figure 3A). For segmental instability, among the 29 patients with grade I spondylolisthesis, no progression was recorded after the procedure. The stabilization effect was sustained and even strengthened as the FLD-EXD (slippage difference between flexion and extension) value gradually decreased during our follow-up period. However, the difference was not statistically significant (*p* = 0.449; Figure 3B).

Changes in the foraminal length, width, and area on both lateral (standing upright) and extension views are summarized in Figure 3C,D. Similar to the disc height findings, all parameters of the intervertebral foramen showed a significant increase in the two different standing positions from the initial postoperative period to the last follow-up (*p* < 0.05). While the foraminal area at the operated level increased, there was no radiographic evidence of adjacent segment degeneration.

### 3.3. MRI and 3D Volumetric Outcomes

**Figure 4:**
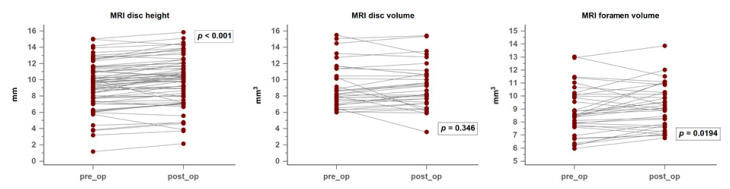
MRI and three-dimensional volumetric changes.

On sagittal T2-weighted MRI, the preoperative disc height at the DIAM-implanted segment increased significantly, with a mean difference of 0.5 ± 0.1 mm (*p* < 0.001). This finding is compatible with what was seen on X-ray radiography while the patients were standing. No progressive intervertebral disc degeneration was reported. For the volumetric evaluation, however, the disc volume calculated from the 3D slicer did not show a similar beneficial effect. The mean volume difference was only 0.21 mm^3^ (−0.33 mm^3^–0.53 mm^3^, 95% CI) (*p* = 0.346). On the other hand, as radiography revealed a significant increase in the foraminal area, the foraminal volume was enlarged after DIAM implantation. The mean foraminal volume difference was 0.4 ± 0.16 mm^3^ (*p* = 0.0194). The changes in MRI disc height and volumetric data are shown in Figure 4.

### 3.4. Correlation between Clinical Symptoms and Image Findings

Nonparametric test statistics and correlation analyses were performed between the preoperative and postoperative groups for clinical outcomes, radiographic outcomes, and 3D volumetric changes. There was a significant correlation coefficient between the Prolo scale and foraminal area calculated from X-ray (*p* = 0.03, r = −0.42) as well as between the Prolo scale and MRI disc volume (*p* = 0.04, r = −0.41). The VAS pain score was not significantly correlated with any imaging findings. The foraminal volume calculated on MRI had no substantial correlation with the Prolo scale or VAS pain score (*p* = 0.11, *p* = 0.09).

### 3.5. Illustrative Case

A 60-year-old female patient presented with low back pain, radicular pain in the left leg, and intermittent neurogenic claudication for 1 year. The symptoms persisted despite medications and physical therapy. The pain was dull but persistent. She could still walk and stand upright in her normal life, but decreased endurance appeared gradually. When visiting our outpatient clinic, her VAS score was 3–5, and the Prolo scale score was 7. Neurologic examination revealed no motor deficits, but she had sciatica on the left side.

**Figure 5:**
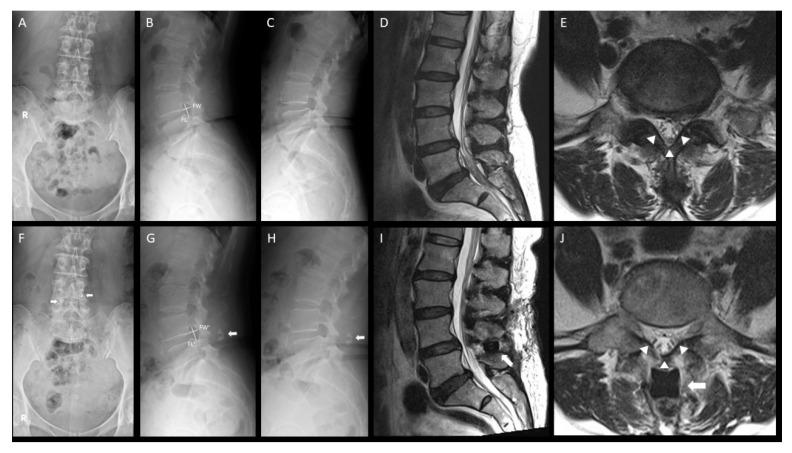
Radiographs and MRI of a 60 year-old-woman with spinal stenosis at L4–5. (**A**–**C**) Preoperative radiographs showed deceased disc height at L4–5 with mild spondylolisthesis. Measurement of Foraminal Length (FL) and Foraminal Width (FW) were demonstrated. (**D**,**E**) MRI showed L4–5 spinal stenosis and dark signal in the disk anterior to left lateral recess. (**F**–**H**) After decompression surgery and DIAM implantation, postoperative radiographs showed increased disc height and preserved segmental mobility. (**I**,**J**) Postoperative MRI showed increased disc height and enlarged neuroforamen (arrowheads). DIAM implants were well fixed postoperatively (arrows in (**F**–**J**)). Abbreviations: FL’ = postoperative foraminal length; FW’ = postoperative foraminal width.

Preoperative radiographs (Figure 5A–C) showed that she had a deceased disc height at L4–5 with mild spondylolisthesis. MRI showed L4–5 spinal stenosis and a dark signal in the disc located anteriorly to the left lateral recess (Figure 5D–E). Foraminotomy with DIAM implantation anchored between the L4 and L5 spinous processes was performed. The patient experienced relief of low back pain and radiculopathy after the surgery and at the 1-year follow-up. However, the newly developed back soreness persisted, which did not affect her regular life. Postoperative radiographs and MRI at 1 year showed an increased disc height and enlarged intervertebral foramen without progression of spondylolisthesis (Figure 5F–J).

## 4. Discussion

The increased prevalence of degenerative lumbar diseases has been well discussed in the past few years as life expectancy has increased. In addition to decompressive lumbar surgery and minimally invasive techniques, implantation of IPDs can act as an adjunct since they are designed under the idea of restoring the foraminal and disc height, unloading axial pressure on the facet joints, and preserving partial sagittal mobility [17]. While Caserta et al. first proposed that DIAM could benefit a certain group of patients suffering from low back pain, the indications for DIAM implantation varied [18]. Several authors have reported high complication and device failure rates if the patient population is not well selected [19,20,21,22]. Generally, the current consensus is that interspinous devices should be preserved for patients with dynamic stenosis, foraminal stenosis, disc herniation, and low-grade segmental instability [3,4].

In our study, patients with low back pain or radiculopathy achieved satisfactory results after DIAM implantation with decompression surgery at the one-year follow-up. Significant correlations between improvements among the VAS and Prolo scale scores were also noted. The short-term outcomes were compatible with those of previous reports [3,4,23]. Low back pain derived from lumbar degenerative diseases has been regarded as multifactorial. Common contributing factors include stimulation of mechanoreceptors on bony structures; nociceptors of soft tissues including the annulus fibrosus and ligaments; and referred pain generated from sinuvertebral nerves [24]. These contributing factors, whenever it is possible that they play a critical role, can be addressed with decompression, laminotomy, or foraminotomy, while preserving all anatomic structures.

In addition to the well-known clinical effects of decompression, the benefits provided by IPDs have been biomechanically proven. In cadaveric studies, Lindsey et al. found that interspinous spacers significantly unloaded intervertebral pressure at the operated level, providing a similar “decompressing” effect, or what some may consider the “sharing” effect [12]. These effects on the posterior functional units then provide less stimulation on the receptors of endplates, joints, soft tissues, and sinuvertebral nerves. Similar effects have been found in vivo, while some studies have further shown that DIAM does not accelerate disc degeneration of adjacent segments because a similar IDP compared to the non-instrumented model was detected [8,13,25].

While the effects of DIAM on low back pain and leg pain have been well discussed in previous literature, patients with soreness failed to improve in our study. The possible mechanisms for this result include foreign body irritation to soft tissue around the treated levels or muscle soreness after posture adjustment due to partially limited ROM in the realigned spine.

In a study of 129 patients with lumbar spinal stenosis, Sobottke et al. found that disc height, foraminal height, foraminal width, and foraminal area significantly increased after implantation of an interspinous device, but these improvements seemed to revert toward preoperative values over time [26]. In our study, the greatest radiographic improvements were all seen at three months postoperatively. Although the effect gradually decreased, it remained sustained during the follow-up period. This is because these instruments were able to maintain the neuroforaminal and canal spaces after partial destruction of the posterior element and distraction of the spinous processes while unloading axial pressure on the facet joints [27]. Our study also recorded changes in the neuroforamen during extension on radiographs because bulging of the ligamentum flavum in this posture could easily cause NIC. The enlarged extensional foraminal area in our study was compatible with the results of previous literature, showing that DIAM can prevent narrowing of the foramina at the instrumented level [17].

Our study showed that the range of motion in flexion and extension at the surgical level decreased after DIAM implantation, although the change was not statistically significant. The results generally correspond to previous interspinous device data, which has been thoroughly studied, both in vitro and in vivo [28,29]. DIAM, in particular, can stabilize unstable segments and reduce implanted levels by 17% in flexion and by 43% in extension [13,30]. Furthermore, when compared to other interspinous devices, DIAM showed the smallest increase at adjacent levels in terms of flexion, lateral bending, and torsion in one biomechanical study [8]. This feature led to its effect in providing a dynamic transition zone while decreasing adjacent segment degeneration after multilevel posterior lumbar interbody fusion [7].

Compared to radiographs, MRI can better evaluate neural structure and volumetric changes. However, studies on MRI changes after DIAM implantation or other interspinous devices are scarce. Siddiqui et al. discovered increased vertebral disc height at the operated level after X stop implantation, but the difference was not statistically significant [31]. Lu et al., on the other hand, found that DIAM could significantly increase the neuroforaminal width and resolution of annular fissures on postoperative MRI, but only in three cases [25]. To our knowledge, our study is the first in the literature to use 3D volumetric assessments to evaluate MRI changes in DIAM implantation. The increase in MRI disc height and foraminal volume suggests that DIAM was capable of preventing disc degeneration and relieving nerve compression. This result was in accordance with the results of previous in vivo studies [8,17]. In contrast, disc volume in 3D assessments did not reach a similar effect at the implanted level. This result could be well explained, as we interpreted biomechanically. The relatively constant disc volume accompanied by increased disc height implies that less axial pressure would lie on the intervertebral disc, providing the aforementioned unloading effect and reducing disc bulging. Through intradiscal pressure measurement, the effect was directly proven [25].

Improvements between clinical outcomes and radiological parameters after IPD implantation showed significant but clinically questionable correlations with the results of previous literature [26]. In our study, we analyzed the correlation among the aforementioned data, MRI disc height, and 3D volumetric changes, in particular. Our results were similar to those of previous studies, showing that improvements in clinical symptoms were not strongly correlated with changes in imaging findings, including multidimensional data calculated from MRI. There are several possible mechanisms for this rather weak correlation. One explanation is that the size of the neuroforamen or disc changes dynamically [32]. While DIAM and other IPDs mostly work on limiting flexion-extension, the less-restricted effects of lateral bending or axial rotation can still alter foraminal and disc volumes. Overall, the 3D volumetric results in our study were still consistent with clinical improvements, proving the clinical benefits of DIAM implantation after decompression surgery.

Our study had several limitations. First, this was a retrospective case study. Possible errors on radiographs or in 3D volumetric assessments may occur. To minimize these potential biases, we enrolled 1 neurosurgeon and 1 neurosurgical resident, who were both blinded to the outcomes, for image evaluation and calculation. Data for statistical analysis were the average values between the two clinicians. Second, although we specifically included patients with low back pain due to degenerative disc diseases or lumbar stenosis, other sources of pain, such as facet pain, could still exist. Third, studies have disclosed high complication, symptom recurrence, and device failure rates after IPD implantation, which were not seen in our study [10,11,22,33]. This could be explained by different surgical techniques since all of our cases undergone decompression surgery; more strictly selected patient groups since we exclude patients with obvious instability, and different texture of IPDs. The other main reason could be the fact that our study had maximum one year of follow-up. In other words, clinical benefit is limited to one year in our study. We hope to provide long-term imaging results and clinical benefits in future works.

## 5. Conclusions

In highly selected patients with low back pain or neurogenic claudication caused by degenerative disc disease, lumbar stenosis, or low-grade spondylolisthesis, three-dimensional assessment showed that limited decompressive surgery with DIAM implantation could enlarge the intervertebral foramen and reduce disc loading, while these improvements were limited postoperatively. The three-dimensional volumetric findings were compatible with the clinical benefits.

## Figures and Tables

**Table 1:** Preoperative characteristics of the patients.

Number	50
Age (years old)	62.1
Gender M/F	13/37
Radiographic diagnosis	
Stenosis	38
Spondylolisthesis	29 (all grade I listhesis)
Herniated disc	15
Levels of operation	
L2–3	4
L3–4	2
L4–5	24
L2–3 and L3–4	3
L3–4 and L4–5	15
L2–3, L3–4 and L4–5	2

F: female; M: male.

**Table 2:** Clinical outcomes.

	Preoperatively	Postoperatively	Improvement Rate
Low back pain	44/50 (88%)	11/50 (22%)	75% *
Leg pain +/− NIC	43/50 (86%)	4/50 (8%)	90.7% *
Soreness	14 (28%)	13 (26%)	7%
VAS (mean ± SE)	3.9 ± 1.7	0.9 ± 1.8	*p* < 0.05
The Prolo Scale (mean ± SE)	6.3 ± 0.9	8.3 ± 1.4	*p* < 0.05

*p* Value < 0.05 was marked asterisk (*), measured under McNemar’s test; NIC = neurogenic intermittent claudication. Questionnaires were given to assess VAS and Prolo Scale. Evaluation of soreness was based on patient’s subjective complaints. SE: standard error.
